# Anæsthesia

**Published:** 1875-01

**Authors:** E. F. Bunnell


THE
AMERICAN JOURNAL
OF
DENTAL SCIENCE.

Vol. VIII. THIRD SERIES--JANUARY, 1875. No. 9.

### ARTICLE I.

### *Anæsthesia.*

BY DR. E. F. BUNNELL.

Read before the California State Dental Association.

The subject of Anæsthesia, both local and general, is one that to a great extent has occupied the minds of surgeons and dentists for many years.

The word anæsthesia indicates an agent that renders a person insensible to pain. It is not my expectation to advance any new ideas upon the subject; it is one that should be, and I presume is, familiar, to you all. My object is simply to induce a discussion upon the merits of the most general articles used by our profession, in order to lead to the adoption of the one that, all things considered, will produce the desired result with the least danger to our patients. It is a subject of great and grave importance to those who are called upon almost daily to perform operations more or less painful, the thought of which produces an unconquerable horror in the minds of very many persons who apply to us for relief. I shall therefore speak of the three substances known as chloroform, sulphuric ether and nitrous oxide. The latter substance, manufactured in a crude form and administered in an imperfect way, was noticed by "Sir Humphrey Davy" about the year 1800; after many experiments he predicted its eventual use for the prevention of pain in surgical operations. To "Horace Wells," a dentist, belongs the credit of verifying this prediction, although its extended use is of comparatively recent date.

No doubt it has, in some cases been greatly abused, in consequence of the imperfection in the apparatus for generating and administering the gas. It has been asserted that it may be used with perfect impunity in all cases, even if the patient is suffering from disease of heart, lungs, or from *any* organic disease. I consider this to be an attempt to prove too much for any substance that will produce perfect anæsthesia. Although the gas is purely oxygen and nitrogen, the element of the air we breathe, yet we are aware that there is great difference between a *mixture* of these elements and a chemical combination.

With this combination it is necessary to exclude atmospheric air—with chloroform and ether this is not admissible, for without air respiration could not be sustained. But as respiration is fully sustained during the administration of nitrous oxide, it proves it to be more nearly normal to atmospheric air. There is doubtless a tendency of blood to the head to some extent during its inhalation. I would therefore consider it unsafe, the same as I would chloroform and ether, where there is a tendency or predisposition to apoplexy or inflammation of the brain.

It has been considered to be one of the laws or effects of stimulation that it is always followed by a corresponding or proportionate depression. This is undoubtedly the case with chloroform and ether, but is not so with nitrous oxide.

The preparation of the gas is very simple, with proper apparatus and proper caution; I would always use a double valve inhaler, attached by a hose of large caliber, directly to the reservoir of gas, so that a free large column may pass directly to the patient. In this way the respiration is free, whereas if the column of gas is small the respiration is more or less labored. An inhaler, with a mouth-piece in the center to pass between the teeth, leaves the mouth open for the operation when anæsthesia is complete.

Now let us examine the components of this substance. It contains only those of atmospheric air, nitrogen and oxygen, chemically combined—the air in its natural state being a *mixture* of nitrogen by weight 76.99, oxygen by weight 23.10, by volume 79.20 and 20.80. By chemical combination of these elements, the proportion is changed to an atmosphere about once and a half the weight of air, and contains nitrogen 63.6 per cent., oxyen 36.4 per cent., or in chemical notation, atoms 1 and 1—equivalent nitrogen 14, oxygen 8.

Anæsthesia is supposed to be produced with it, by supply ing the system with carbon more rapidly than it can be eliminated. The patient passes quickly into a perfect state of anæsthesia, which is always plainly indicated. The con dition is of shorter duration than that produced by chloroform or ether. The functions of the body are slightly exalted, and respiration fully supported. After the lapse of from two to five minutes, the patient is in as perfectly a normal condition as before inhaling it. It is generally believed that any substance that produces immunity from pain is followed by a corresponding penalty. This I believe is not the case with nitrous oxide, but we all know it to be more or less so when chloroform or ether is inhaled. We can only judge of the merits of each by actual experiment upon a great variety of temperaments and conditions, considering also the component parts of each and their general effects. It has been asserted that the gas effects only the nerves of motion, and that the patient suffers all the pain without the ability to move. In some cases this may be so. It is equally true that the reverse is more generally the case, the sensory nerves being affected to a greater extent than the motor nerves; the former may occur from one of two reasons anly—if the gas is impure, or deteriorated by the admission of atmospheric air, that condition may occur.

That many fatal results have followed the inhalation of chloroform, no one pretends to deny. It will not do for us to say that we have administered it many thousand times without accident or detriment to our patients, for we may not be aware of the condition produced by its administration upon the nervous system, whereby the health may be impaired for life.

Again it is a well established fact that persons have inhaled it many times with apparent impunity, and death has been finally produced before perfect anæsthesia has been obtained, and this too when no reasonable cause should be assigned. Statistics show us that these results have more frequently happened with persons in perfect health, so far as relates to any organic disease, than otherwise. In a table published in the *New York Journal of Medicine*, about the year 1864, it is stated that three died instantly, two in a minute, ten in from two to ten minutes, one in a quarter of an hour, one in half an hour, one in three hours, and the remainder in periods varying slightly from those above mentioned, to the number of thirty-three. Up to that date the recorded list of fatal results ascribed to chloroform amounted in the aggregate to more than one hundred. Now taking it for granted that we are willing to take the chances of producing death during an operation, what have we to fear beyond this?

Chloroform—or properly, ter-chloride of fomyl—contains 12 parts carbon, 1 part hydrogen, and 10.5 parts chlorine. When inhaled it is rapidly transformed in the blood, producing *formic acid*, which acts upon it as a poison; and having a strong affinity for the blood, it may remain in the circulation sufficiently long to produce effects upon the system which may and often do prove highly injurious. One thing is certain, the poison is transmitted to the circulation, and if anæsthesia is produced, it is no matter by whom or how carefully it is administered, the poison must be there.

Chloroform was discovered by Samuel Guthrie, of Sackets Harbor, N. Y. Its chemical constituents were ascertained by "Dumas" in 1834. In its general influence it is sedative primarily to the nervous system; and secondarily, to respiration and circulation. It is absorbed by the blood and has been detected both in the blood and tissues after death. If pushed beyond the stage of comma and complete muscular relaxation, respiration becomes diminished, the pulse feeble, the face livid; finally respiration ceases, and also the heart's action—death being the result. When a very small quantity has been inhaled, death has been known to ensue by local paralysis of the heart, and in some cases respiration has continued after the heart has ceased to beat.

In regard to the after effects of the two substances—chloroform and ether—although the latter as well as the former produces languor, attended often with nausea and other disagreeable consequences, sometimes lasting for days, I would assuredly give the preference to ether. The component parts of the latter are: 24 parts carbon, 8 parts oxygen, and 5 parts hydrogen. When inhaled, it is decom posed in the system, forming *acetic acid* in the circulation, which is neutralized or passes off by after breathing, leaving no injurious substance in the blood.

During the administration of nitrous oxide, it has been suggested that the purple appearance of the lips indicate a tendency to asphyxia. This cannot be true, for the reason that *respiration* is fully supported.

Statistics recently collated by Professor E. Andrews, published in the *Chicago Medical Examiner*, gives the following estimate of the relative danger in the administration of different anæsthetics; in 200,893 cases, viz:

Sulph. ether, one death in 23,204 administrations.

Chloroform, one death in 2,723 administrations.

Mixed chloroform and ether, one death in 5,588 administrations.

Bi-chloride of methylene, one death in 7,000 administrations.

*Nitrous Oxide*, no deaths in 75,000 administrations.

I do not wish to be understood to favor anæsthetics of any kind, to be used generally in our practice; yet I believe there are cases where it is admissible to use other than local anæsthetics. I believe these cases are few, and that proper discrimination should be made when they are used. I simply desire to give the preference over chloroform and ether to nitrous oxide, when either is admissible.

N. B.—Some portions of the foregoing I do not claim to be original, they having been taken from publications that have appeared upon the subject; I have used such as best express my views.

